# The impact of COVID-19 pandemic restrictions on offline and online grocery shopping: New normal or old habits?

**DOI:** 10.1007/s10660-022-09658-1

**Published:** 2022-12-26

**Authors:** Philipp Brüggemann, Rainer Olbrich

**Affiliations:** FernUniversität in Hagen, Universitätsstraße 11, 58097 Hagen, Germany

**Keywords:** New normal, Old habits, Online grocery shopping, Pandemic restrictions, Purchase pattern, Retail

## Abstract

The COVID-19 pandemic is changing future trends in retailing and e-commerce immensely. Recent research revealed a considerable increase in online grocery shopping (OGS) since the COVID-19 pandemic started. In addition, current statistics indicate a steady increase in OGS over the coming years. Despite this, less is known about whether consumers’ behavior is evolving to a ‘new normal’ or returning to ‘old habits’ after pandemic restrictions are withdrawn. To address this research gap, we operationalize and empirically analyze offline and online purchasing behavior before, during, and after pandemic restrictions. To this end, we use an extensive household panel dataset of 17,766 households reporting their purchases before, during and after the first lockdown in Germany in 2020. Our findings on offline purchase patterns show that while more than 10% of the consumers avoided brick-and-mortar retail during the lockdown, almost all of them returned afterwards. Looking at online purchase patterns, we find high volatility in OGS for both separate and combined purchase patterns. The combined analysis of purchase patterns (online and offline), reveal that households that avoided brick-and-mortar stores during the lockdown did not switch (completely) to the online channel. Based on our findings that consumers are still in reach of brick-and-mortar retailers we suggest offline retailers act now to retain their customers, e.g., by offering competitive benefits in their stores. OGS operators should urgently analyze the customer churn revealed in this analysis and derive measures to retain them. They do not seem to have succeeded in retaining their customers and keeping them loyal to the online channel during the entire observation period. Even worse, they also failed to convince consumers to use OGS who stayed at home due to the lockdown. The fact that a total of 96.75% of the observed consumers did not practice OGS at all shows that OGS in Germany was in 2020 still in its infancy. However, as current statistics forecast a further substantial increase in OGS over the coming years, our results are increasingly relevant for brand managers, brick-and-mortar retailers and OGS providers in Germany and beyond.

## Introduction

The COVID-19 pandemic has changed the world of retailing dramatically over a short period of time [1]. On the one hand, these changes pose immense challenges for brand managers and retailers. On the other hand, it also provides new opportunities for retailing and e-commerce [2]. For instance, consumers’ habits have been strongly restricted by governmental policies, especially by lockdowns. Brick-and-mortar retail was limited to the most necessary during this period. While grocery stores predominantly stayed open, other retail sectors did not. The resulting decline in sales and even insolvencies of entire businesses will change whole business sectors [3–5]. These transformations will, in turn, also affect the future of grocery shopping, e.g., in increasing opportunities to buy groceries online [6–8]. While the consequences of the COVID-19 pandemic are dramatically and existentially challenging, it also creates scope for new practices and emerging business models. Roggeveen and Sethuraman [1] suggest that online grocery shopping (OGS) will increase as a result of the COVID-19 pandemic. Additionally, they expect changes in consumer behavior in the future. Recent statistics support this expectation, as worldwide data show an increase in OGS turnover, especially since the COVID 19 pandemic [9]. Furthermore, latest forecasts based on these data point to a continuous increase in OGS over the coming years [9].

Within the COVID-19 pandemic OGS offers new opportunities to gain competitive advantages [10]. However, companies need to know how to act on these developments. Despite its high relevance, there is still a lack of research in analyzing different consumers’ purchase patterns before, during, and after pandemic restrictions. In particular, understanding the impact of pandemic restrictions (e.g., lockdown) on consumer buying behavior is important to help brand managers and retailers rapidly respond to these immense changes. In fact, “ignoring trends can give rivals the opportunity to transform the industry” [11].

Numerous studies have already confirmed the increase in use of OGS during the COVID-19 pandemic worldwide [12–22]. Additionally, Brüggemann and Pauwels [23] found significant differences between also-online and offline-only grocery shoppers in both consumers’ attitudes and purchase behavior. However, it has not yet been investigated how offline and online grocery purchases are affected by pandemic restrictions on household level. Verhoef et al. [24] synthesize empirical and conceptual research on effects of COVID-19 on retailing. The authors state that COVID-19 changes consumer needs and behavior and that retailers need to know how to respond. In addition, the authors conclude that more structured methods should be used to examine future trends in OGS due to the pandemic. With our research, we provide such a structured method for analyzing changes in consumer behavior. Taken toghether, we identify a lack of research on whether a ‘new normal’ will emerge or whether consumers will return to ‘old habits’ in both offline and online grocery shopping.

For instance, consumers who were already familiar with OGS before COVID-19 pandemic may also shop online during and after pandemic restrictions. Furthermore, it is also conceivable that consumers who previously purchased offline-only might also shop online during pandemic restrictions, but return to their old habits afterwards. Conversely, some consumers might continue to purchase online after pandemic restrictions, at least to some extent.

With this research we provide new insights on how consumers behave before, during, and after pandemic restrictions, both when shopping for groceries offline and online. For this purpose, we use household panel data from 2020. We observe offline and online purchases before, during, and after the first lockdown of the COVID-19 pandemic in Germany from March 22, 2020 to May 5, 2020. We examine changes in offline and online purchases of 17,766 households by conducting structured analyses of household purchasing behavior. This approach provides for the first time sophisticated insights into how consumers’ offline and online grocery shopping behavior evolved before, during, and after pandemic restrictions. Taken together, we address the following research question:

How does consumer purchase behavior evolve during and after pandemic restrictions in terms of online and offline grocery shopping?

## Theoretical background

### Covid-19 and pandemic restrictions

The COVID-19 virus first emerged in China in December 2019 and has spread rapidly throughout the world. The virus has killed over 1.6 million people and sickened over 76 million people [25]. In Germany the first COVID-19 case was detected in January 2020. To limit the spread of the virus, multiple interventions have been put in place. In addition to mandatory masks and social distancing, temporary lockdowns were imposed. The first lockdown in Germany was implemented from March 22, 2020 to May 5, 2020. During this time people were forced to stay at home. Only with a valid reason (e.g., for work, grocery shopping, or doctor’s appointment) it was allowed to leave one’s home. The supermarkets remained open during the lockdown to ensure households’ supply [26].

**Fig. 1:**
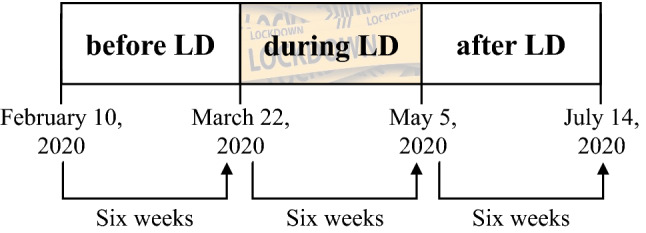
Periods before, during, and after pandemic restrictions

Figure 1 shows the three time intervals in 2020 that are relevant for this study. In previous research on purchase patterns before, during, and after pandemic restrictions, Brüggemann and Olbrich [27] utilized data from two years. Here, the observed time periods are of different lengths. On the one hand, this also includes households that purchase irregularly. On the other hand, there is a risk that these households randomly did not shop during the lockdown. Therefore, in this research we consider periods of equal length for the periods before, during, and after the lockdown. Based on this, we define the first observation period from February 10, 2020 to the start of the first lockdown on March 22, 2020. The second period includes the first lockdown, which lasted until May 5, 2020. Finally the third interval considers the period after the lockdown until July 14, 2020. As a result, we obtain three observation periods, each of six weeks in length.

German consumers were able to shop in brick-and-mortar stores during the entire observation period. Given the unchanged accessibility of those stores, we are particularly interested in how this lockdown nevertheless affect grocery shopping, especially with regard to the choice between online and offline channels. By differentiating the three observation periods, we can investigate how resilient households are to changes in distribution channels. To investigate this in a well-founded manner, we first review the relevant literature on grocery retailing in the following section.

### Grocery retailing

Grocery retailing, as well as research in the field, has a long tradition [e.g., 28–30]. However, retailing is still constantly evolving. According to Guha et al. [31], the increased use of artificial intelligence will transform commerce, e.g., in order to increase both in-store and online sales, to improve the effectiveness of supply chains, or to make the payment more efficient. As well as current technological developments, the COVID-19 pandemic is also a driver of change. For example, Roggeveen and Sethuraman [1] expect that the COVID-19 pandemic will cause consumers to become accustomed to new ways of shopping, such as OGS and home delivery. In addition, the authors state that people may be more likely to work at home in the future and thus be more likely to book online sports courses or to buy an in-home bike, for example. Overall, a major change in consumer behavior when purchasing groceries is expected as a result of the COVID-19 pandemic [32, 33].

Both the accelerating development and the use of new technologies in retail are also playing increasingly important roles in the channel structure. Whereas a few decades ago products were sold almost mainly via brick-and-mortar stores or catalogs, consumers have become accustomed to online ordering and home delivery. This development is especially facilitated by technical progress. Well-known examples of large e-commerce companies in the retail sector are Amazon and Alibaba [34].

For grocery shopping, the online channel has also been discussed in numerous publications over the past decades [35–41]. However, before the COVID-19 pandemic, the development of OGS was slow [42–44]. There is consensus in the literature that COVID-19 has accelerated this development, at least short-term [13, 45, 46]. However, it is not implied that this strong increase in OGS will continue after the COVID-19 pandemic.

According to East [47], it is likely that the post-pandemic increase in share of OGS will return to pre-pandemic levels, especially for discount purchasing. Gruntkowski and Martinez [48] found that the COVID-19 pandemic led to a reduction of consumers expected risk regarding OGS. Furthermore, Tyrväinen and Karjaluoto [49] used a meta-analysis to examine OGS before and after the COVID-19 pandemic. They find that perceived usefulness and attitudes before the COVID-19 pandemic have a strong influence on intention to use OGS. The authors conclude that the increasing adoption of OGS is not due to higher expected usefulness or more positive attitudes, but that consumers were driven to use OGS by the COVID-19 pandemic. Despite this extensive meta-analysis, the results are limited and further research is needed. For example, the meta-analysis data is based only on questionnaires, not on purchase data. In addition, the authors did not identify any publication that looked at the same households both before and after COVID-19 restrictions.

Shen et al. [46] investigate pandemic effects on OGS usage as well. They studied online purchase patterns before, during, and after COVID-19. The authors identified a sharp increase in OGS use among consumers during the COVID-19 pandemic. After the pandemic, consumers still report a higher use of OGS than before, though Shen et al.’s [46] data suggests a decrease in usage, especially in the long-term. However, these results are limited, too. The findings are merely based on survey data, and most of the respondents are habitants of the same city in the United States.

### Research framework and contribution

After shedding light on previous literature, we find that a broad analysis of online and offline grocery shopping that considers equal periods before, during, and after pandemic restrictions with real purchase data does not yet exist. Nevertheless, it is enormously relevant for companies to obtain information on changes in consumer preferences and buying behavior in online and offline channels at an early stage in order to respond accordingly.

With this study, we address this research gap by examining purchases by households before, during, and after the first lockdown in Germany. Our contribution is to identify different purchase patterns online and offline before, during, and after the first lockdown in Germany in order to determine whether different consumer groups react differently to such restrictions. We additionally broaden our contribution by looking at the online and offline purchase patterns combined. This allows us to examine how the same household behaves online and offline before, during and after the pandemic. In the next sections, we provide information about the data operationalization and descriptive statistics and describe the resultung purchase patterns.

## Empirical analysis

### Data operationalization and descriptive statistics

We use household panel data from 2020 provided by the *GfK*.^1^ The data includes purchases from the product groups chocolate bars, coffee, hair shampoo, and laundry detergent from more than 30,000 German households. The market research institute *GfK* compiles their household panel according to several criteria (e.g., household size, household location, social status, nationality, number of children,…). Thus, they provide a representative data set for German households with regard to these criteria [50]. Moreover, it is recorded whether a purchase were made offline or online. Online and offline channels can belong to the same retailer or to different retailers. We use this differentiation because we are particularly interested in the decision of households to shop offline or online.

**Fig. 2:**
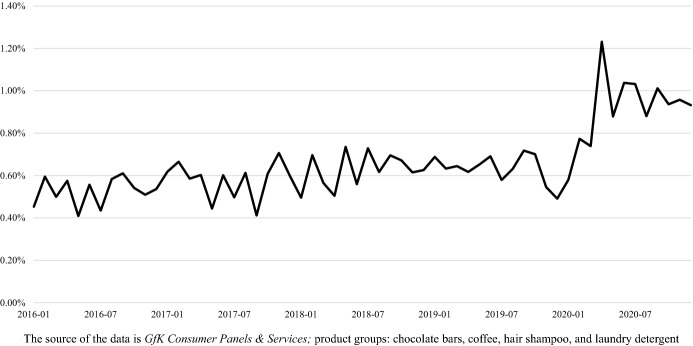
Volume based share of OGS per month (calculation by the authors)

Figure 2 shows the volume-based share of OGS per month based on the household panel data we use for this empirical analysis. Here, we find a comparatively strong increase in the volume-based share of OGS during the lockdown. However, the figure also shows that after the first lockdown the volume-based share of OGS decreases. Overall, the volume-based share of OGS seems to be higher after the lockdown than before. The information provided by this figure is interesting but limited. We cannot see here if/how the households’ purchase patterns change before, during, and after the lockdown. This is where our empirical analysis comes in.

We analyze the purchase data under consideration of the first lockdown in Germany from March 22, 2020 to May 5, 2020. To allow for a reliable analysis, we consider identical periods before, during, and after the lockdown. Since the lockdown lasted six weeks, we consider the purchases six weeks before and six weeks after this lockdown (see Fig. 1). Additionally, only households that purchased both before and after the lockdown are included in the analysis to handle the problem of panel mortality. We can thus observe 17,766 households reporting their offline and online purchases during the eighteen-week observation period. It should be emphasized that this reduction of the basic sample (from more than 30,000 households to 17,766 households) may affect the representativeness regarding all German households described above.

Table 1 provides some key figures for total purchases (offline and online) as well as for offline and online purchases separately. First of all, it can be noted that we examine purchases over a period of six weeks in each period. In the case of total purchases (offline and online), it is noticeable that some households have not made any purchases during the lockdown (from 17,766 to 15,873). The quantity sold, volume and value fell slightly during the lockdown, but slightly rose again afterwards. Value per quantity decreased slightly during the lockdown, but also increased to the pre-lockdown level after the lockdown. Overall, value per kilogram is even higher after the lockdown than before. The offline purchases show a very similar picture, not least because they also largely determine the total purchases due to their high proportion.

**Table 1:** Descriptive statistics about key performance indicators

Channel	Key performance indicator	Before lockdown	During lockdown	After lockdown
Offline and online	Observation period	Six weeks	Six weeks	Six weeks
	Households	17,766	15,873	17,766
	Quantity	127,022	120,653	120,609
	Volume	42,914.99 kg	38,496.37 kg	39,177.17 kg
	Value	285,414.30 €	266,641.41 €	272,999.30 €
	Value/quantity	2.25 €/piece	2.21 €/piece	2.26 €/piece
	Value/kg	6.65 €/kg	6.93 €/kg	6.97 €/kg
Offline	Observation period	Six weeks	Six weeks	Six weeks
	Households	17,722	15,828	17,691
	Quantity	126,113	119,320	119,311
	Volume	42,633.06 kg	38,105.96 kg	38,822.55 kg
	Value	281,789.72 €	261,458.99 €	268,249.55 €
	Value/quantity	2.23 €/piece	2.19 €/piece	2.25 €/piece
	Value/kg	6.61 €/kg	6.86 €/kg	6.91 €/kg
Online	Observation Period	Six weeks	Six weeks	Six weeks
	Households	208	284	283
	Quantity	909	1,333	1,298
	Volume	281.93 kg	390.41 kg	354.62 kg
	Value	3,624.58 €	5,182.42 €	4,749.75 €
	Value/quantity	3.99 €/piece	3.89 €/piece	3.66 €/piece
	Value/kg	12.86 €/kg	13.27 €/kg	13.39 €/kg

Looking at online purchases, we see that the number of households buying online increases during the lockdown (from 208 to 284) and remains almost at this level after the lockdown (283). The quantity sold, volume and value show that after the increase during the lockdown, value decrease slightly again. Overall, these descriptive data indicate a moderate increase in OGS due to the lockdown. Interestingly, the value per quantity and the value per kilogram decrease during this period. Compared to offline purchases, it can be seen that both value per quantity and value kilogram are considerable higher online than offline. In order to be able to consider product group-specific differences in the later discussion of the empirical results, we also provide information on the structure of the data in terms of the four product groups we use. Table 2 shows the shares of the four product groups in the data we use.

**Table 2:** Descriptive statistic about the proportions of the product groups used

		Quantity			Volume			Value		
		Before lockdown (%)	During lockdown (%)	After lockdown (%)	Before lockdown (%)	During lockdown (%)	After lockdown (%)	Before lockdown (%)	During lockdown (%)	After lockdown (%)
Offline and online	Laundry detergent	6.65	5.23	6.24	33.58	28.63	31.20	14.58	11.99	13.63
	Hair shampoo	7.56	6.33	7.09	6.78	6.07	6.64	7.63	6.72	7.28
	Chocolate bars	53.04	54.68	53.17	19.16	20.95	19.70	22.77	24.15	22.73
	Coffee	32.75	33.76	33.50	40.49	44.34	42.47	55.01	57.14	56.36
	Sum	100.00	100.00	100.00	100.00	100.00	100.00	100.00	100.00	100.00
Offline	Laundry detergent	6.65	5.24	6.23	33.58	28.65	31.23	14.66	12.10	13.73
	Hair shampoo	7.55	6.26	7.03	6.77	6.01	6.57	7.62	6.64	7.19
	Chocolate bars	53.26	55.03	53.49	19.24	21.07	19.79	22.98	24.49	23.01
	Coffee	32.53	33.47	33.25	40.41	44.27	42.41	54.75	56.77	56.08
	Sum	100.00	100.00	100.00	100.00	100.00	100.00	100.00	100.00	100.00
Online	Laundry detergent	6.49	4.05	6.86	33.50	26.63	27.43	8.63	6.30	8.08
	Hair shampoo	8.58	12.30	12.71	7.68	12.48	14.24	8.81	10.95	12.75
	Chocolate bars	21.34	23.56	23.81	7.05	9.49	9.60	6.96	6.94	7.33
	Coffee	63.59	60.09	56.63	51.76	51.40	48.74	75.60	75.81	71.84
	Sum	100.00	100.00	100.00	100.00	100.00	100.00	100.00	100.00	100.00

For total purchases (offline and online) and offline purchases, the shares are mostly stable before, during and after the lockdown, with the exception of coffee. For coffee, the data suggest a slight increase in the share of coffee in the data during and after the lockdown. For online purchases, the share of coffee is surprisingly relatively high. Here, a positive trend can be seen during and after the lockdown. This also partly explains the significantly higher value per quantity and value per kilogram as coffee is comparatively more expensive than chocolate bars, hair shampoo, and laundry detergent. Furthermore, a positive trend can also be seen in the data for online purchases of hair shampoo. These findings must be considered when interpreting the results. In the next section, we present the operationalization of the purchase patterns.

### Purchase patterns

**Fig. 3:**
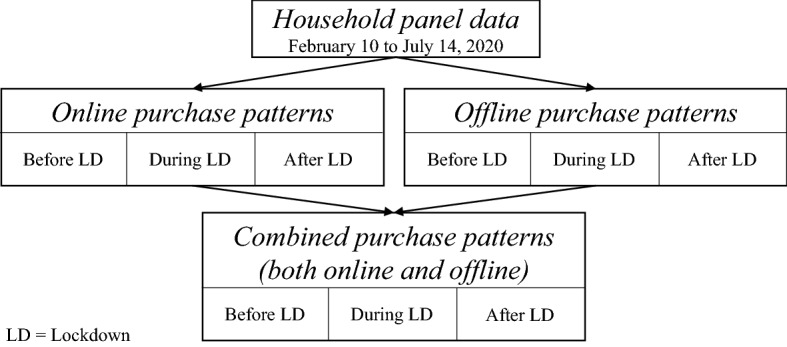
Online, offline and combined purchase patterns

Figure 3 shows the procedure for separating the household panel data to calculate online, offline, and combined purchase patterns. In the first investigation, we separate households between online and offline purchases, derive online and offline purchase patterns, and analyze them separately, respectively for the three periods before, during, and after the lockdown. In the second investigation, we combine selected offline and online purchase patterns to observe the behavior of households online and offline simultaneously.

**Fig. 4:**
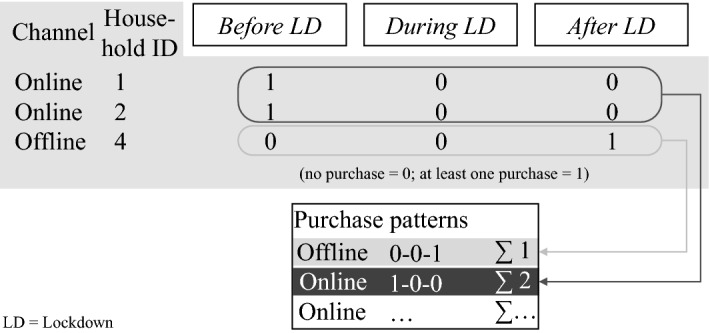
Calculation of online and offline purchase patterns

For both offline and online purchases, we derive eight purchase patterns to observe consumers’ purchase behavior before, during, and after the lockdown. To identify the different purchase patterns, we code them by attributing to each household ‘0’ for no purchases and ‘1’ for at least one purchase in each observation period (before, during, and after lockdown). For instance, a coding of 1-0-0 means that the related household only purchased before the lockdown and neither during nor after. Figure 4 illustrates the calculation of purchase patterns using three exemplary purchases from three different households.

Using this procedure, we obtain eight online and eight offline purchase patterns. The following Table 3 provides an overview and detailed descriptions of the 16 resulting purchase patterns. In the next sections, the empirical findings on online and offline purchase patterns are examined separately. This is followed by an empirical analysis of the combined purchase patterns.

**Table 3:** Offline and online purchase patterns

Channel	Purchase pattern	Description
Offline	Offline 1-0-0	No more offline shoppers since the lockdown
	Offline 0-1-0	Offline shoppers during the lockdown
	Offline 0-0-1	New offline shoppers after the lockdown
	Offline 1-0-1	Non-offline shoppers during the lockdown
	Offline 0-1-1	Offline shoppers since the lockdown and beyond
	Offline 1-1-0	No more offline shoppers after the lockdown
	Offline 1-1-1	Continuous offline shoppers
	Offline 0-0-0	Online-only shoppers
Online	Online 1-0-0	No more online shoppers since the lockdown
	Online 0-1-0	Online shoppers during the lockdown
	Online 0-0-1	New online shoppers after the lockdown
	Online 1-0-1	Non-online shoppers during the lockdown
	Online 0-1-1	Online shoppers since the lockdown and beyond
	Online 1-1-0	No more online shoppers after the lockdown
	Online 1-1-1	Continuous online shoppers
	Online 0-0-0	Offline-only shoppers

### Empirical findings

#### Offline purchase patterns

The offline purchase patterns before, during, and after the lockdown are shown in Table 4. Based on these purchase patterns, different descriptions are specified (see second column). The majority of consumers purchased offline before, during, and after the lockdown (88.88%; 15,777). During the lockdown, 10.65% (1890) did not purchase offline. The fact that these consumers return to brick-and-mortar retail after the lockdown shows that they (at least partially) return to their ‘old habits’ and do not completely change their behavior to OGS.

**Table 4:** Offline grocery purchase patterns

Purchase pattern	Household types	Quantity	Share of offline grocery shopping (%)	Share of total grocery shopping (%)
Offline 1-0-0	No more offline shoppers since the lockdown	27	0.15	0.15
Offline 0-1-0	Offline shoppers during the lockdown	5	0.03	0.03
Offline 0-0-1	New offline shoppers after the lockdown	6	0.03	0.03
Offline 1-0-1	Non-offline shoppers during the lockdown	1,890	10.65	10.64
Offline 0-1-1	Offline shoppers since the lockdown and beyond	18	0.10	0.10
Offline 1-1-0	No more offline shoppers after the lockdown	28	0.16	0.16
Offline 1-1-1	Continuous offline shoppers	15,777	88.88	88.80
Sum offline		17,751	100.00	99.92
Offline 0-0-0	Online-only shoppers	15		0.08
Sum total		17,766		100.00

1 = purchases, 0 = no purchases; with purchases ‘before-during-after’ lockdown; n = 17,766

#### Online purchase patterns

Table 5 shows the results regarding online purchase patterns. Here, the empirical results show a much more differentiated behavior than in offline purchasing behavior. Among the (also-)online purchasing households, 18.86% (109) purchased online only before the lockdown (‘online 1-0-0’). Exclusively during the lockdown, 29.58% (171) of (also-)online grocery shoppers purchased online (‘online 0-1-0’). In other words, nearly one-third of these households started shopping online during the first lockdown and stopped afterwards. Thus, the lockdown appears to have been a reason for these households to try OGS. However, they did not continue to use OGS afterwards.

**Table 5:** Online grocery purchase patterns

Purchase pattern	Household types	Quantity	Share of online grocery shopping (%)	Share of total grocery shopping (%)
Online 1-0-0	No more online shopping since the lockdown	109	18.86	0.61
Online 0-1-0	Online shoppers during the lockdown	171	29.58	0.96
Online 0-0-1	New online shoppers after the lockdown	150	25.95	0.84
Online 1-0-1	Non-online shoppers during the lockdown	35	6.06	0.20
Online 0-1-1	Online shoppers since the lockdown and beyond	49	8.48	0.28
Online 1-1-0	No more online shoppers after the lockdown	15	2.60	0.08
Online 1-1-1	Continuous online shoppers	49	8.48	0.28
Sum online		578	100.00	3.25
Online 0-0-0	Offline-only shoppers	17,188		96.75
Sum total		17,766		100.00

1 = purchases, 0 = no purchases; with purchases ‘before-during-after’ lockdown; n = 17,766

After the lockdown, 25.95% (150) of (also-)online grocery shoppers chose OGS for the first time (‘Online 0-0-1’). On the one hand, this suggests that a lockdown increases the number of online shopping households only in the short term. On the other hand, there still is a substantial amount of fluctuation among OGS. Only a few households purchased (also-)online before and after the lockdown, but not during (6.06%; 35) (‘Online 1-0-1’). Other consumers started using OGS (8.48%; 49) (‘Online 0-1-1’) or stopped using it since the lockdown (2.60%; 15) (‘Online 1-1-0’). Only 8.48% (49) of (also-)online purchasing households bought groceries online before, during, and after the lockdown (‘Online 1-1-1’). In comparison with offline purchases, the (also-)online purchasing households represent only 3.25% (578).

Taken together, more than half of online shoppers try OGS, but then buy offline only again (51.04%; 295).^2^ Furthermore, almost all of the observed households shop offline (again) after the lockdown (99.58%; 17,691).^3^ While 35.99% (208)^4^ of the observed (also-)online shopping households shopped groceries online before the lockdown, 48.96% (283)^5^ shopped for groceries online afterwards. Overall, our results of the online grocery purchase patterns show strong dynamics in the use of OGS.

#### Combined purchase patterns

Beyond the separated analyses of offline and online purchase patterns, this section looks at combined purchase patterns. This allows us to observe how the households behave both online and offline before, during, and after pandemic restrictions. Regarding offline purchase patterns, the previous investigation suggests, that two patterns are almost solely relevant. Thus, in the further analysis, we focus on these two offline purchase patterns.

First, we consider households that made offline purchases continuously throughout the observation period (‘offline 1-1-1’). These households comprise 88.88% (15,777) of offline purchasing households. Second, we consider households that did not shop at brick-and-mortar stores during the lockdown (‘offline 1-0-1’). These households comprise 10.65% (1890) of offline buying households.

The previous results have revealed strong dynamics in the use of OGS. Therefore, in the further analysis we consider all eight online purchase patterns and combine each of them with the two offline purchase patterns mentioned above (‘offline 1-1-1’ and ‘offline 1-0-1’). Thus, sixteen combined purchase patterns emerge. The combined purchase patterns, their descriptions, quantity and share are shown in Table 6.

**Table 6:** Combined purchase patterns

Offline purchase pattern	Online purchase pattern	Household types	Quantity	Share of total grocery shopping (%)
1-0-1	1-0-0	No more online shopping since lockdown, while not shopping offline during the lockdown	7	0.04
1-0-1	0-1-0	Switching from offline to online during the lockdown	12	0.07
1-0-1	0-0-1	Starting online shopping after the lockdown, while not shopping offline during the lockdown	14	0.08
1-0-1	1-0-1	Not shopping groceries during the lockdown at all	5	0.03
1-0-1	0-1-1	Switching from offline to online during the lockdown, shopping both online and offline afterwards	6	0.03
1-0-1	1-1-0	No more online shopping after the lockdown, while not shopping offline during the lockdown	0	0.00
1-0-1	1-1-1	Continuous online shoppers, who stopped shopping offline during the lockdown	4	0.02
1-0-1	0-0-0	Offline-only shopping households before and after the lockdown	1842	10.37
1-1-1	1-0-0	No more online shoppers since lockdown, while continuous offline shopping	89	0.50
1-1-1	0-1-0	Online shoppers during the lockdown, while continuous offline shopping	159	0.89
1-1-1	0-0-1	New online shoppers after lockdown, while continuous offline shopping	109	0.61
1-1-1	1-0-1	Non-online shoppers while lockdown, while continuous offline shopping	15	0.08
1-1-1	0-1-1	Online shoppers since lockdown and beyond, while continuous offline shopping	27	0.15
1-1-1	1-1-0	No more online shoppers after lockdown, while continuous offline shopping	11	0.06
1-1-1	1-1-1	Continuous both online and offline shoppers	21	0.12
1-1-1	0-0-0	Offline-only purchasing households	15,346	86.38
Missing			99	0.56
Sum			17,766	100.00

For offline purchase patterns, only 1-1-1 and 1-0-1 patterns are considered. The offline purchase patterns not considered include 99 households (see “missing”)

The combination of purchase patterns shows that the majority of households shop groceries steadily offline and not at all online. This segment comprises 86.38% (15,346) of the observed households (‘offline 1-1-1 and online 0-0-0’). The second largest segment consists of households that did neither shop in brick-and-mortar stores nor online groceries during the lockdown (‘offline 1-0-1 and online 0-0-0’). This group comprises 10.37% (1842) of all observed households. This means that even the households that did not shop offline during the lockdown also predominantly did not switch to the online channel. This shows that the lockdown hardly caused households to switch from offline to online.

Only 0.89% (159) of the households used OGS during the lockdown and neither before nor after (‘offline 1-1-1 and online 0-1-0’). However, these households use OGS only in a complementary way, as they continued to shop offline.

Furthermore, 0.61% (109) of the observed households started shopping online after the lockdown (‘offline 1-1-1 and online 0-0-1’). However, these households continue to shop offline as well. The empirical results show that 0.50% (89) of the observed households have not shopped online since the lockdown (‘offline 1-1-1 and online 1-0-0’). These households have thus stopped using OGS during the observation period and have returned to purchase exclusively offline. Interestingly, only 0.12% (21) households made both online and offline purchases in each period (‘offline 1-1-1 and online 1-1-1’). Further purchase patterns with comparatively small numbers of cases can be seen in Table 6. In the next chapter we will discuss the implications of our findings.

## Implications

### Offline purchase patterns

The results of this study clearly show that German grocery shopping habits we observed differ between offline and online channels. Regarding offline purchase patterns, we found that almost all of the observed households purchase groceries from brick-and-mortar retailers after the lockdown (99.58%; 17,691).^6^ It is particularly relevant for retailers that 10.65% (1890) of the observed households avoided brick-and-mortar retail stores during the lockdown, but returned to buy groceries in brick-and-mortar stores afterwards. Hence, offline grocery shopping is mainly affected by a lockdown in the short term and consumers are still in reach of brick-and-mortar retailers. Our household panel data only show a comparatively strong increase in the share of OGS during the lockdown (see Fig. 2). Moreover, current forecasts for Germany and worldwide show a substantial increase in OGS over the coming years, at least until 2027 [9, 52]. This indicates a risk that offline retailers will increasingly lose market shares to online grocery retailers. Thus, brick-and-mortar retailers should now focus on generating and communicating competitive advantages over online providers (e.g., via price, experience, or service) as long as this is feasible for them. For instance, consumers can haptically experience the products in brick-and-mortar retail, which is hardly possible in online stores [52].

After all, if retailers lose households to online grocery retailers, influencing and winning back these consumers will be much more challenging. Offline retailers should also think about adding an online channel in order to reduce their disadvantages compared to online providers (e.g., shopping at anytime and anywhere as well as home delivery).

### Online purchase patterns

Compared to the offline purchase patterns, we found considerably more divergent online purchase patterns. Since 51.04% (295)^7^ of (also-)online purchasing consumers bought groceries online before or during the lockdown but not afterwards, OGS providers should critically evaluate their customer retention measures. OGS providers need to identify these consumers as well as possible reasons for their suspended OGS use.

However, we also find consumers who started using OGS during the observation period. For instance, 34.43% (199)^8^ of (also-)online grocery shoppers began shopping for groceries online during or after the lockdown. This high fluctuation presents both threats and opportunities for brand managers as well as for retailers. For instance, there is a risk that actual customers will either switch to competitive online suppliers or satisfy their needs in brick-and-mortar stores (again). The high dynamic of OGS can also persuade new households to shop for groceries online. The fact that more than 96% of households made purchases exclusively offline throughout the observation period shows that OGS was not very widespread in Germany in 2020. However, this shows the immense untapped potential for OGS. As current forecasts predict a further increase in the share of OGS in grocery retailing over the coming years, both in Germany and worldwide [9, 51], OGS providers should work now on improving customer loyalty in order to benefit from this trend in the long term.

### Combined purchase patterns

The combined purchase patterns reveal that households who avoided brick-and-mortar stores during the lockdown hardly switched to OGS. Thus, 10.37% (1842) of all households neither shopped offline nor started OGS during the lockdown. This means that almost none of these households started buying groceries online during or after the lockdown (0.11%; 20).^9^ For OGS providers, this shows how tightly households are still holding on to their ‘old habits’ instead of trying new ways of shopping. Therefore, this insight is relevant for both OGS providers and brick-and-mortar retailers.

Online grocery shop operator need to understand that changing consumer habits is a process that often can only be accomplished over an extended period of time. Hence, OGS providers are faced with the difficulty to persuade households to try OGS. Targeted incentives should be created and communicated, e.g., welcome gifts or additional benefits such as automatic shopping lists or deposit acceptance. The high volatility in the use of OGS should be considered by the providers of OGS in their strategy to attract additional customers. On the one hand, households must be convinced of the use of OGS and its advantages through communication (e.g., time savings due to home delivery). On the other hand, however, the customers acquired must also be tied to OGS in the long term. It seems the latter has not been successful during the observation period, since many households discontinue OGS.

For brick-and-mortar retailers, the data indicate that the households are sticking to or returning to their ‘old habits’. Moreover, our results for the combined purchase patterns reinforce the results of the separate purchase patterns. Offline retailers still have access to households through their stores, given that almost all customers at least returned to offline shopping during the observation period. Retailers need to act now and convince customers of the benefits of brick-and-mortar stores in the future. The results of the combined purchase patterns clearly show that households do not immediately switch to the online channel even during a lockdown. Therefore, the need for brick-and-mortar stores is demonstrated and their existence justified. Furthermore, as OGS become more widespread worldwide [51], the pressure on brick-and-mortar stores will increase. For this reason, retailers should pay particular attention to customer loyalty.

## Concluding remarks

### Summary

This study contributes to a better understanding of how consumer purchasing behavior evolves during and after pandemic restrictions on online and offline grocery shopping. During the first lockdown in Germany, 10.37% (1842) of the consumers avoided brick-and-mortar stores. The offline purchase patterns are characterized by ‘old habits’, since after the lockdown almost all of the observed consumers visit brick-and-mortar stores again. Thus, consumers did not completely switch from offline to online channel as a result of pandemic restrictions. Our results provide valuable insights for brick-and-mortar retailers, at least in Germany. Our empirical results indicate that it is still possible for retailers to influence customers in their own stores, since almost all of the consumers observed still shop (also-)offline. Particularly against the backdrop of the predicted increase in OGS over the coming years in Germany and worldwide [9, 51], brick-and-mortar retailers should now develop strategies to retain their customers in the long term. They can seek to do this, for example, by gaining competitive advantages over online retailers (e.g., through price, experience, or service) or by adding an online channel. If consumers are accustomed to online channels and increasingly purchase their groceries online, it will become much more challenging for brick-and-mortar retailers to reach out to these consumers. Fundamentally, it is key for these companies to anticipate and act on these game-changing trends to shape new standards and be successful in the long term [11]. While our 2020 data for Germany show a high momentum in OGS with still relatively low shares of OGS (see Fig. 2), the general trend in Germany and worldwide points to an increase in OGS in the coming years [9, 51, 53]. As a result, the competitive pressure on brick-and-mortar stores will continue to rise, making it particularly necessary for them to improve customer retention.

Our findings on OGS show an ongoing process of change due to a high level of dynamism in online purchase patterns. During the observation period, the buying behavior of (also-)online purchasing consumers changes in different ways. Some consumers started OGS before or during the lockdown, but then switched back to brick-and-mortar stores. Other consumers started OGS before or during the lockdown and maintained this shopping behavior afterwards. These findings indicate a ‘new normal’–at least for certain consumer segments.

Looking at combined online and offline purchasing behavior, we find that households not using offline stores during the lockdown did not switch to the online channel. So there is no entire shift from the offline to the online channel due to pandemic restrictions. Moreover, we also find high volatility in OGS among combined purchase patterns. Online grocery shop operators do not seem to have succeeded in retaining their customers during the observation period. Even worse, they also failed to convince consumers to use OGS who stayed at home due to the lockdown.

In conclusion, this study provides valuable insight into consumer purchasing behavior in Germany both offline and online before, during, and after pandemic restrictions. In particular, brand managers and retailers should take these findings to consider changes in consumers’ behavior in more detail in order to derive measures to increase loyalty. In addition, these new insights provide deeper knowledge of consumer behavior between offline and online channels, as well as consumer responses to crises.

Additionally, the empirical results indicate that it will be crucial for offline retailers to retain their customers, since those who have switched completely to OGS can hardly be reached by offline retailers without enormous efforts. Fortunately, however, we have for now hardly found any households that exclusively use the online channel to purchase groceries.

For online grocery shop operators, it is key to not only continue to attract new customers, but to retain existing customers. The possibly high acquisition costs for new customers (e.g., due to ads, welcome gifts, or discounts) are pointless if customers are not bound to the provider in the long term. In this case the provider will not be able to compete in the long term.

This research differs from previous publications in several aspects. While recent publications, e.g., by Tyrväinen and Karjaluoto [49], Gruntkowski and Martinez [48], Shen [46], Younes et al. [54], Gomes and Lopez [55] and Eriksson and Stenius [56] are based on survey data, we provide an empirical analysis based on real purchase data. This allows us to observe purchases of the same households before, during and after the pandemic to show the evolution of offline and online grocery shopping. Morever, to the best of our knowledge, this research is the first analyzing offline and online channel choices before, during, and after the pandemic using an extensive dataset. In terms of content, we provide several new insights into how German households’ behavior changed differently online and offline in 2020.

### Limitations and further research directions

Even this study has some limitations. First, it cannot be clearly proven that the changes in the purchasing behavior of offline and online purchase patterns are caused by the COVID-19 pandemic. The processes of change in consumer purchasing behavior may be also driven (at least in part) by digitization, increasing online offers, and changing demands.

Second, we cannot draw a detailed picture of shifts in demand between offline and online purchases. Looking at Fig. 2, we can see a comparatively strong increase in the still low volume-based market share of OGS during the lockdown. Moreover, Table 1 shows an increase in the number of online shopping households, quantity sold, volume and value over time. However, while this study focuses on consumer behavior in terms of offline and online channel use, further research should, for example, analyze volume-based purchase patterns in more detail.

Third, Table 2 reveals some differences in the proportions of the observed product groups between online and offline channels. Furthermore, Table 2 shows, for example, that before, during, and after the lockdown, online purchases of coffee increased proportionately. Further research should therefore analyze how online and offline grocery shopping develops differently in terms of product groups and what impact this evolution has on channel performances. Thus, further research may run cross-sectional analyses to reveal differences between products groups.

Fourth, the data in this analysis are exclusively from German households. However, further analysis should go beyond that and compare the results with data from other countries. Thus, further research should complement both cross-national and country-specific insights to OGS research.

Fifth, our data only covers the period up to 2020. Recent statistics show that OGS turnover in Germany increased by 21.21% from 2020 to 2021. Interestingly, the data from 2021 to 2022 shows a slight decrease in OGS turnover of 1.14%. From 2022 to 2027, the annual growth rate in Germany is predicted to exceed on average 15.85% [51]. Worldwide, a very similar annual turnover growth rate of 14.92% is predicted from 2021 to 2027 [9]. Overall, these key figures show the very dynamic development of OGS both for Germany and worldwide. Moreover, these key figures underline the relevance of this topic, for both online grocery store operator and brick-and-mortar retailer. Based on this, we expect our results to be highly relevant for Germany and beyond. However, in further research, the development of online and offline grocery shopping need to be analyzed with more recent data.

Finally, further research should investigate whether consumers with different purchase patterns differ, e.g., in terms of consumer characteristics and demographics, attitudes towards price consciousness or brand preference as well as purchase behavior and attitudes towards organic or fair trade products.

## Footnotes

1 The source of the data is *GfK Consumer Panels & Services*.

2 Considered purchase patterns: ‘Online 1-0-0’, ‘Online 0-1-0’, and ‘Online 1-1-0’.

3 Considered purchase patterns: ‘Offline 0-0-1’, ‘Offline 1-0-1’, Offline 0-1-1- ‘, and ‘Offline 1-1-1’.

4 Considered purchase patterns: ‘Online 1-0-0’, ‘Online 1-0-1’, ‘Online 1-1-0’, and ‘Online 1-1-1’.

5 Considered purchase patterns: ‘Online 0-0-1’, ‘Online 1-0-1’, Online 0-1-1- ‘, and ‘Online 1-1-1’.

6 Considered purchase patterns: ‘offline 0-0-1’, ‘offline 1-0-1’, ‘offline 0-1-1’, and ‘offline 1-1-1’.

7 Considered purchase patterns: ‘online 1-0-0’, ‘online 0-1-0’. and ‘online 1-1-0’.

8 Considered purchase patterns: ‘online 0-0-1’ and ‘online 0-1-1’.

9 Considered purchase patterns: ‘offline 1-0-1 and online 0-0-1’ and ‘offline 1-0-1 and online 0-1-1’.
